# Supplementary material for: Gene networks under circadian control exhibit diurnal organization in primate organs
Source: Commun Biol. 2022 Jul 29;5:764. doi: 10.1038/s42003-022-03722-0 (PMC9334736; doi:10.1038/s42003-022-03722-0)
Supplement: Supplementary file 1 — Supplementary information [file 42003_2022_3722_MOESM1_ESM.pdf]

Supplementary Information

Gene networks under circadian control exhibit diurnal organization in primate organs

Jie Li,[1,#] Pengxing Nie,[1,#] Christoph W. Turck[2] and Guang-Zhong Wang [1,*]

[1]CAS Key Laboratory of Computational Biology, Shanghai Institute of Nutrition and Health, University of Chinese Academy of Sciences, Chinese Academy of Sciences, Shanghai 200031, China
[2]Max Planck Institute of Psychiatry, Proteomics and Biomarkers, Munich 80804, Germany
[#] These authors contributed equally to this work.

* E-mail: guangzhong.wang@picb.ac.cn (G-Z. Wang)

This PDF file includes:

Supplementary Fig. 1 to 17

Other supplementary materials for this manuscript include the following:
Supplementary Code

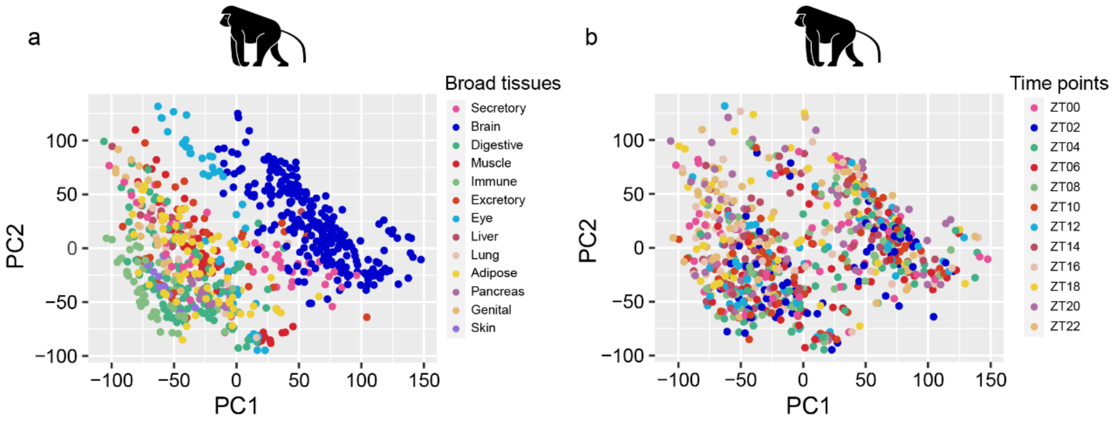

Supplementary Fig. 1 Principal component analysis of baboon diurnal transcriptome atlas. **a** 13 tissue samples are shown in different colors. **b** All samples are clustered for 12 circadian time points.

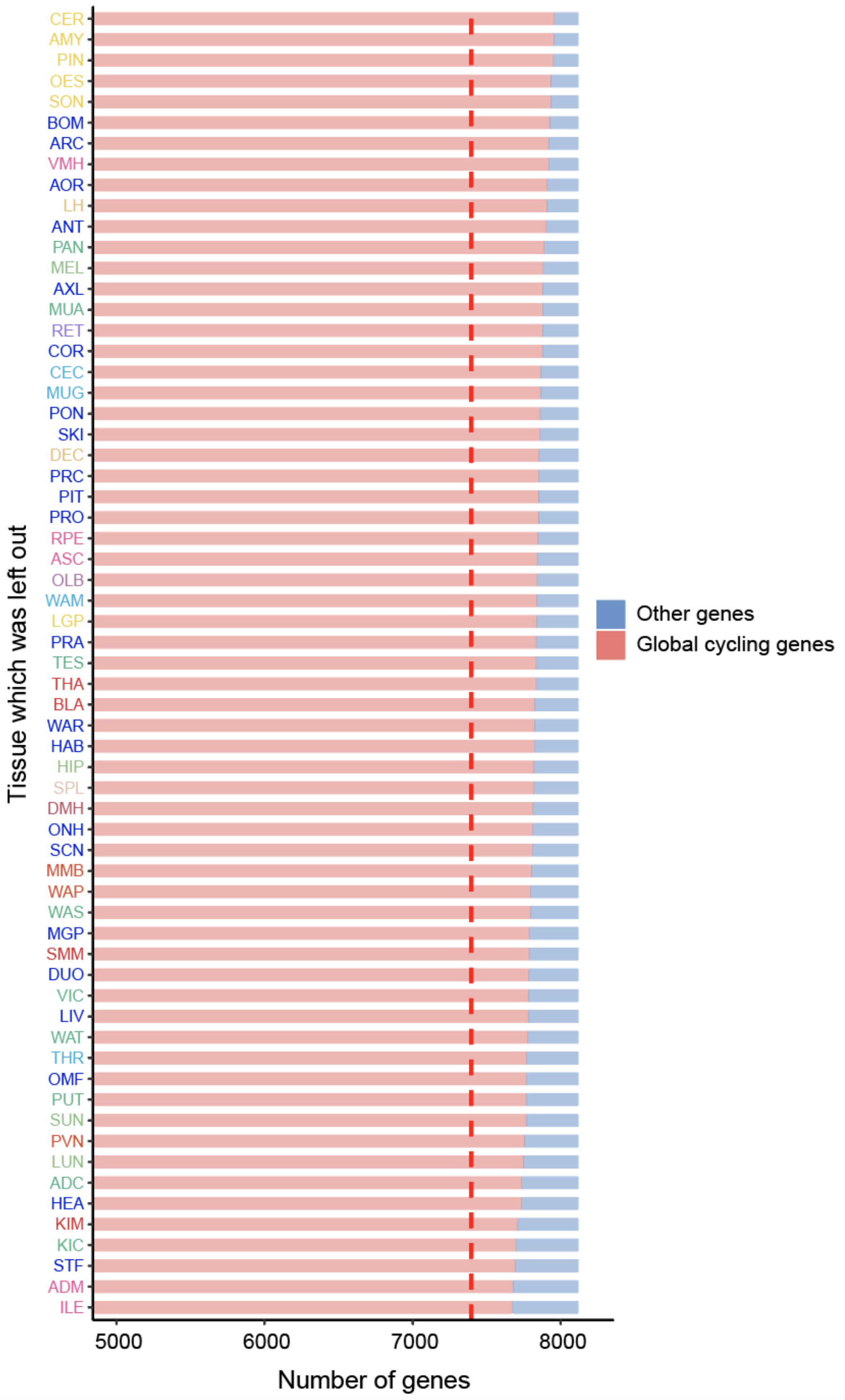

Supplementary Fig. 2 Global cycling genes can be robustly identified in leave one out cross validation tests. In each test, we removed one organ and performed the identification of global cycling gene. Dotted line represents the number of global cycling genes that can be detected in all the tests.

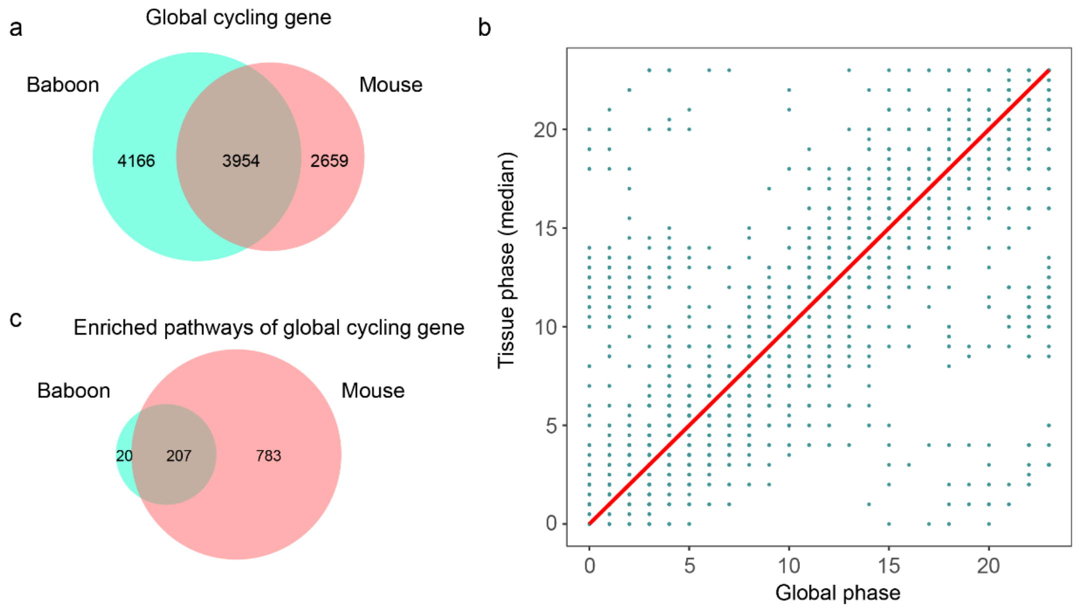

Supplementary Fig. 3 Global cycling genes are identified in both baboon and mouse. a Venn diagram shows the overlap of global cycling genes between baboon and mouse. **b** The phase of global cycling genes correlates with the median phase of cycling genes in 12 mouse organs. **c** Venn diagram shows the number of shared function terms enriched in baboon and mouse.

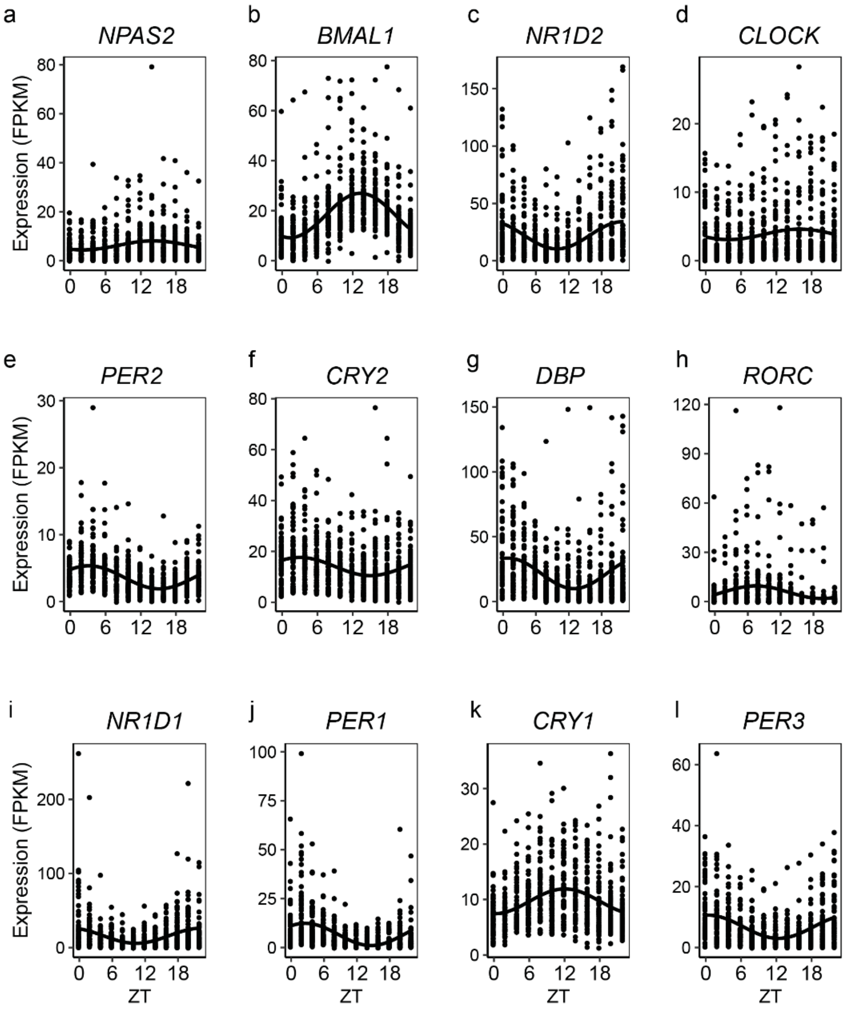

Supplementary Fig. 4 The oscillation of core clock genes across whole primate body. a - l Expression curve over different time points is obtained by nonlinear fitting of cosine model.

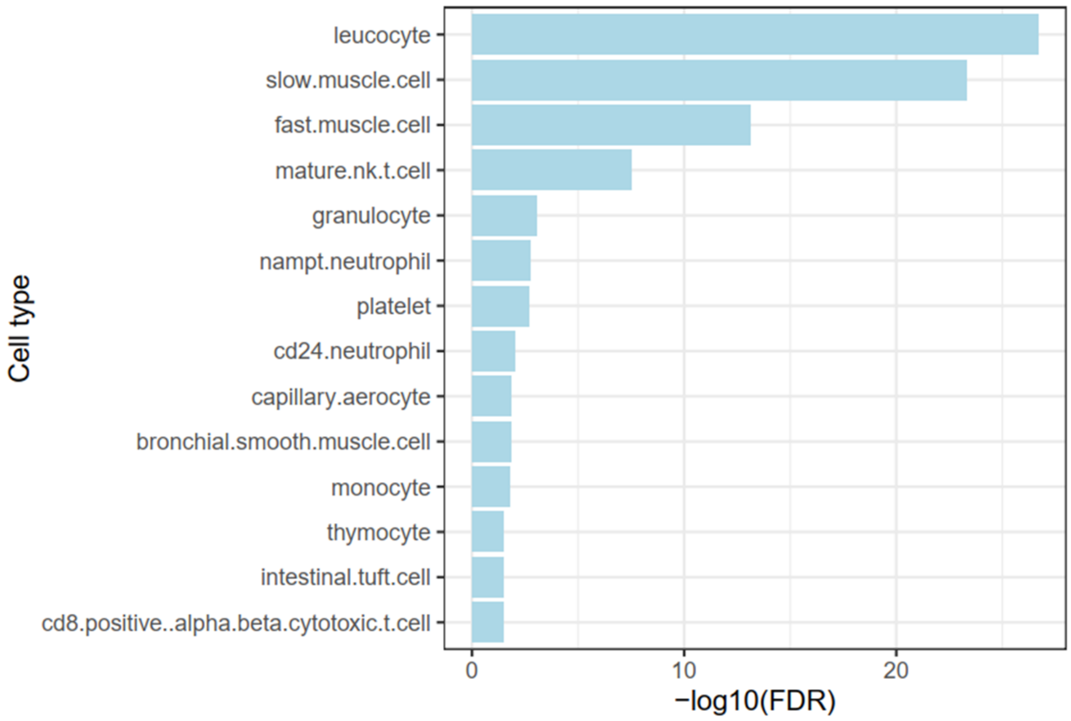

Supplementary Fig. 5 Overrepresented cell types of global cycling genes in Tabula Sapiens.

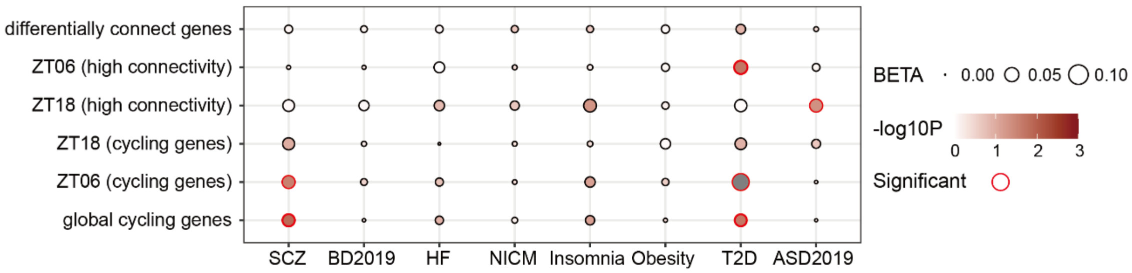

Supplementary Fig. 6 MAGMA analysis shows enrichment of disease risk genes in global cycling genes. The bubble size corresponds to effect size (BETA) and is colored by enriched p value (-log10). SCZ, schizophrenia; BD, bipolar disorder; HF, heart failure; NICM, nonischemic cardiomyopathy; T2D, type 2 diabetes; ASD, autism spectrum disorder.

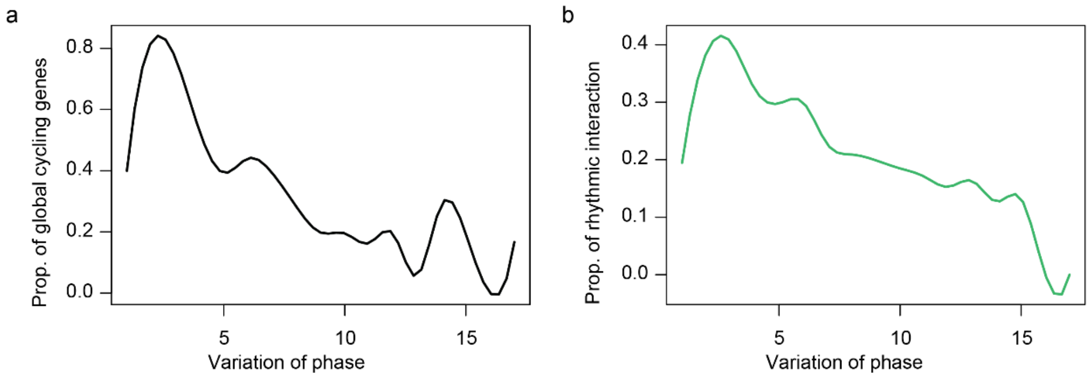

Supplementary Fig. 7 High variation of phase between 63 baboon tissues negatively correlates with the proportion of global cycling genes and rhythmic interaction. a Relationship between proportion of global cycling genes and variation of phase of circadian genes in individual tissues. **b** Relationship between proportion of rhythmic interaction and variation of phase of circadian genes in individual tissues

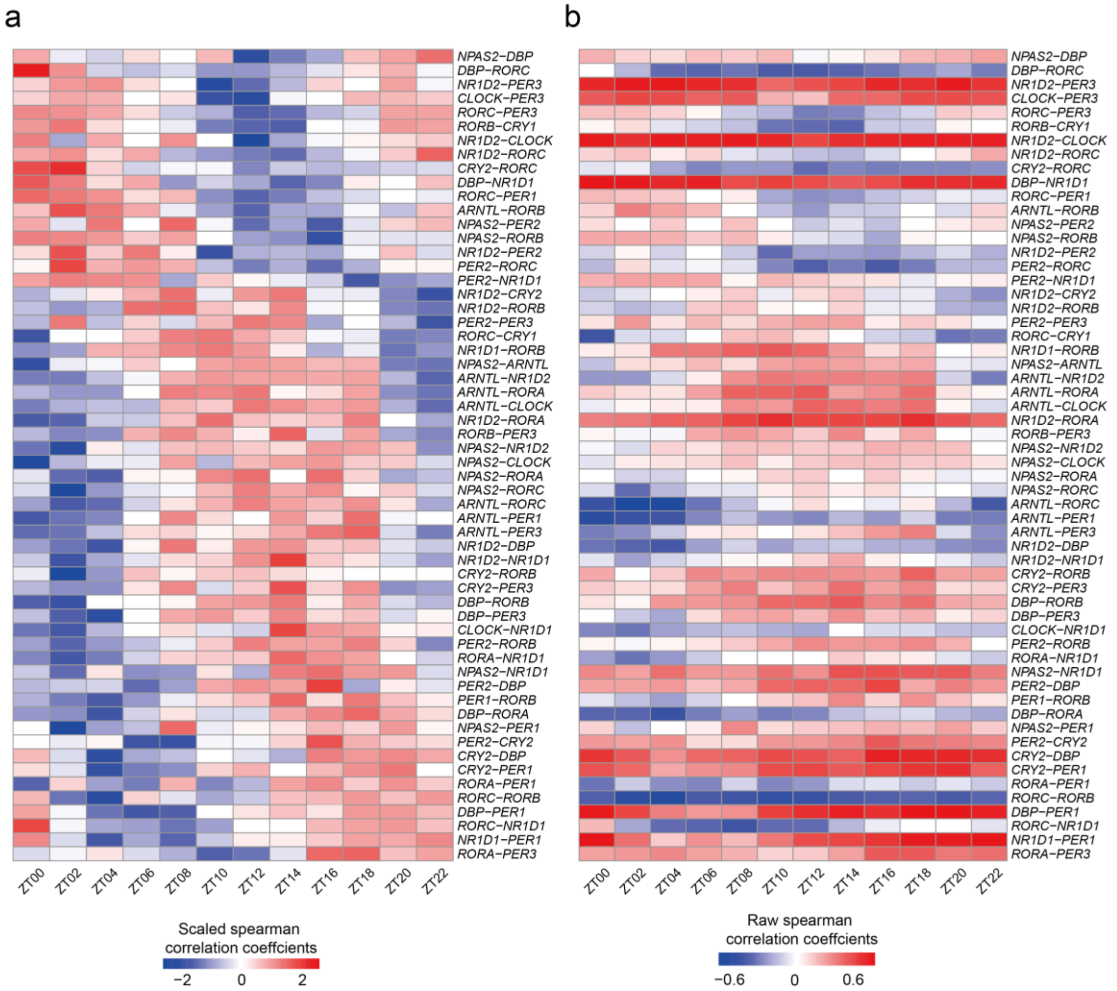

Supplementary Fig. 8 Rhythmic co-expression interactions among core circadian genes. The heatmap represents the strength of the interaction between core circadian gene pairs for both the scaled (a) and raw spearman correlation coefficients (b).

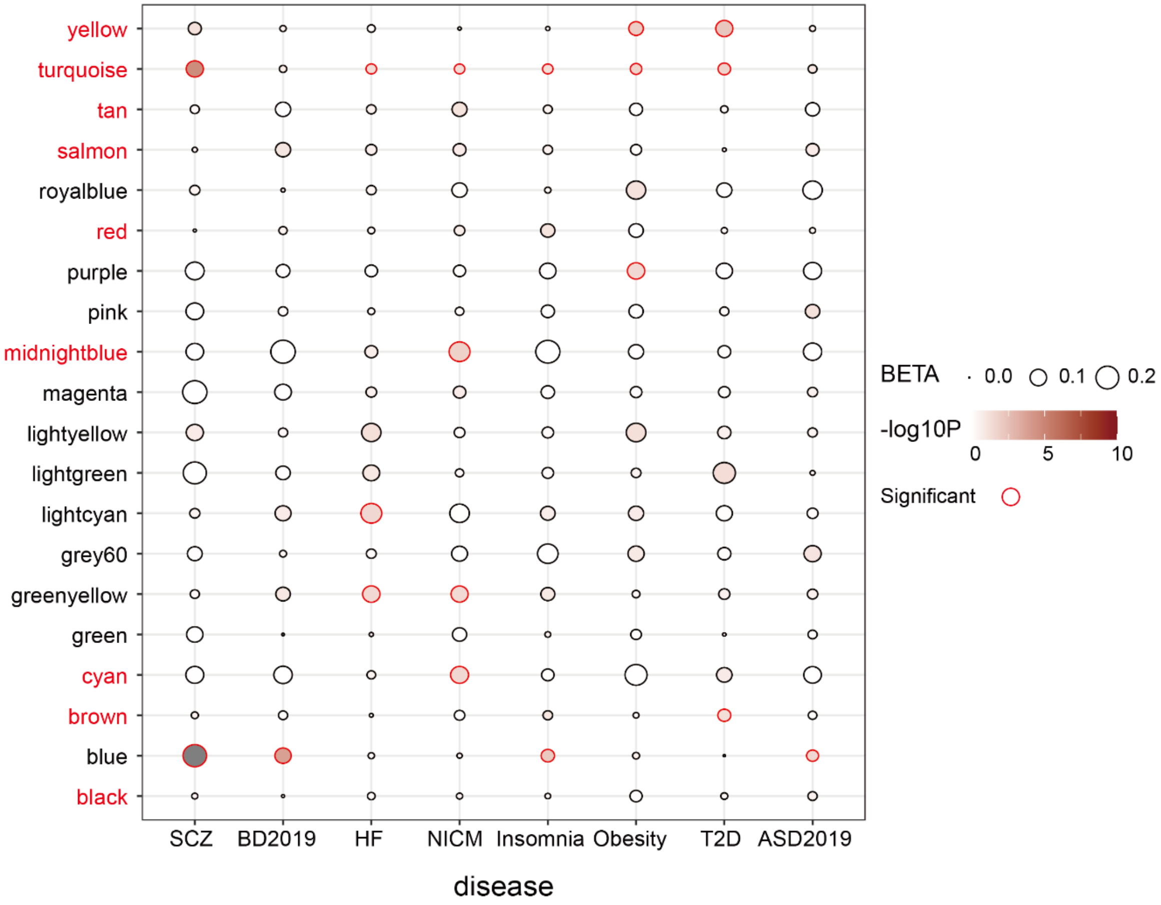

Supplementary Fig. 9 MAGMA analysis shows enrichment of disease risk genes in co-expression modules. The bubble size corresponds to effect size (BETA) and is colored by enriched p value (-log10). Cycling modules are labeled in red. SCZ, schizophrenia; BD, bipolar disorder; HF, heart failure; NICM, nonischemic cardiomyopathy; T2D, type 2 diabetes; ASD, autism spectrum disorder.

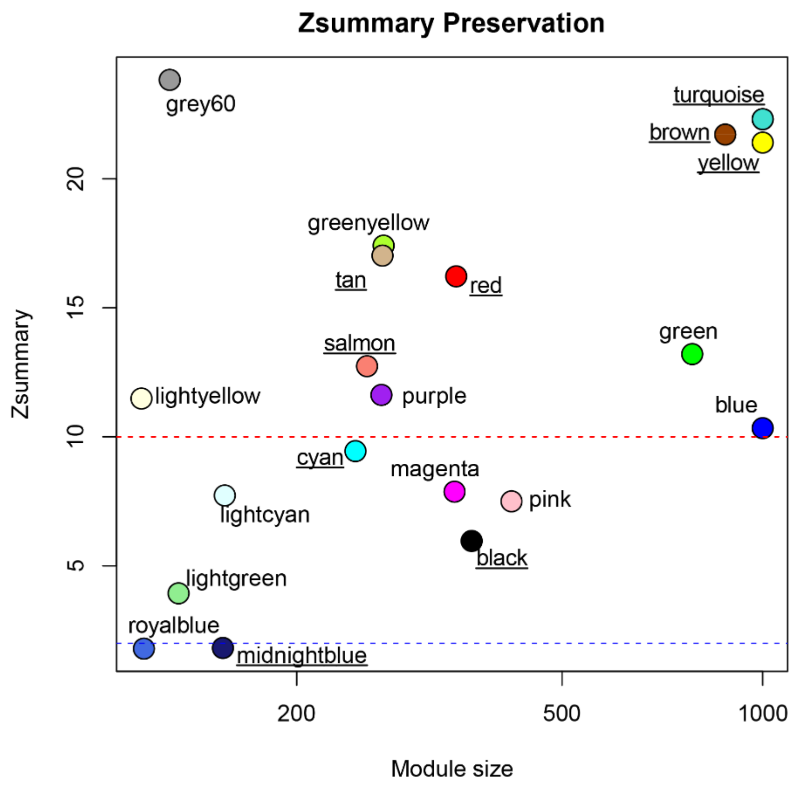

Supplementary Fig. 10 Module preservation between baboon and mouse cycling transcriptome. Cycling modules are underscored. The blue dotted line represents Zsummary = 2 and the red dotted line Zsummary = 10.

a

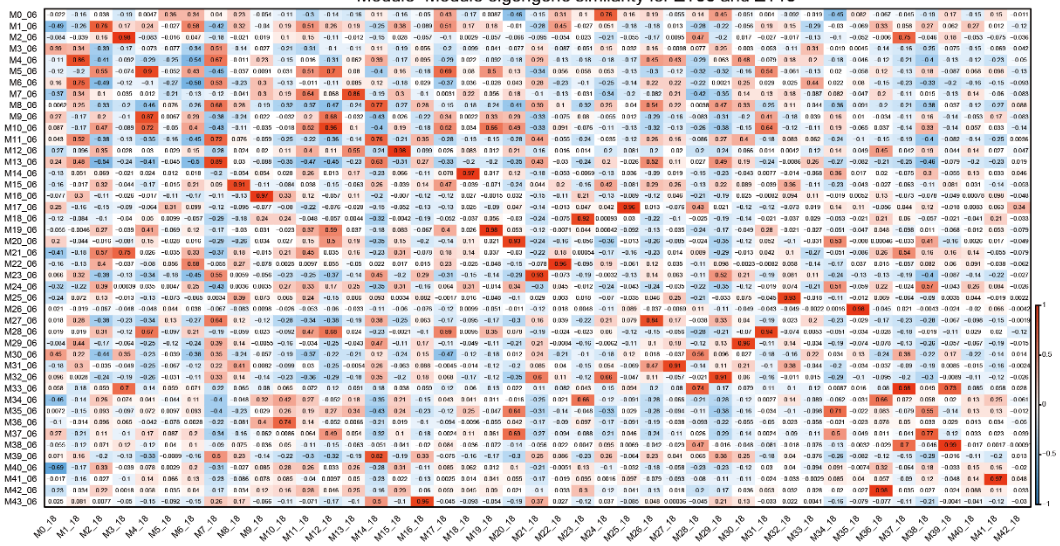

b

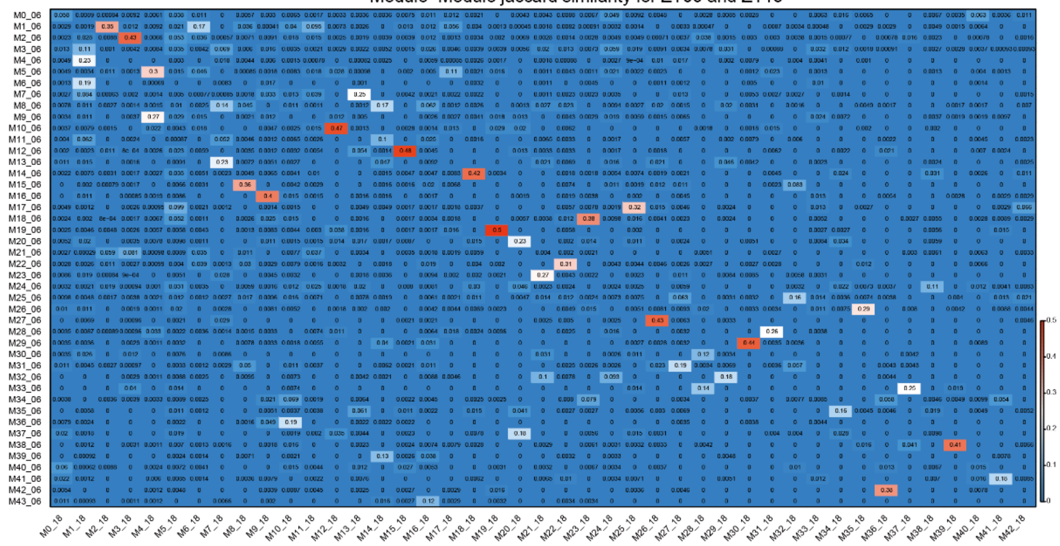

Supplementary Fig. 11 Similarity between the ZT06 (noon) and ZT18 (night) modules. a Eigengene similarity among different modules. The module eigengene was calculated by WGCNA. **b** Jaccard similarity among different modules. The jaccard similarity measures to what extent two modules include the same genes.

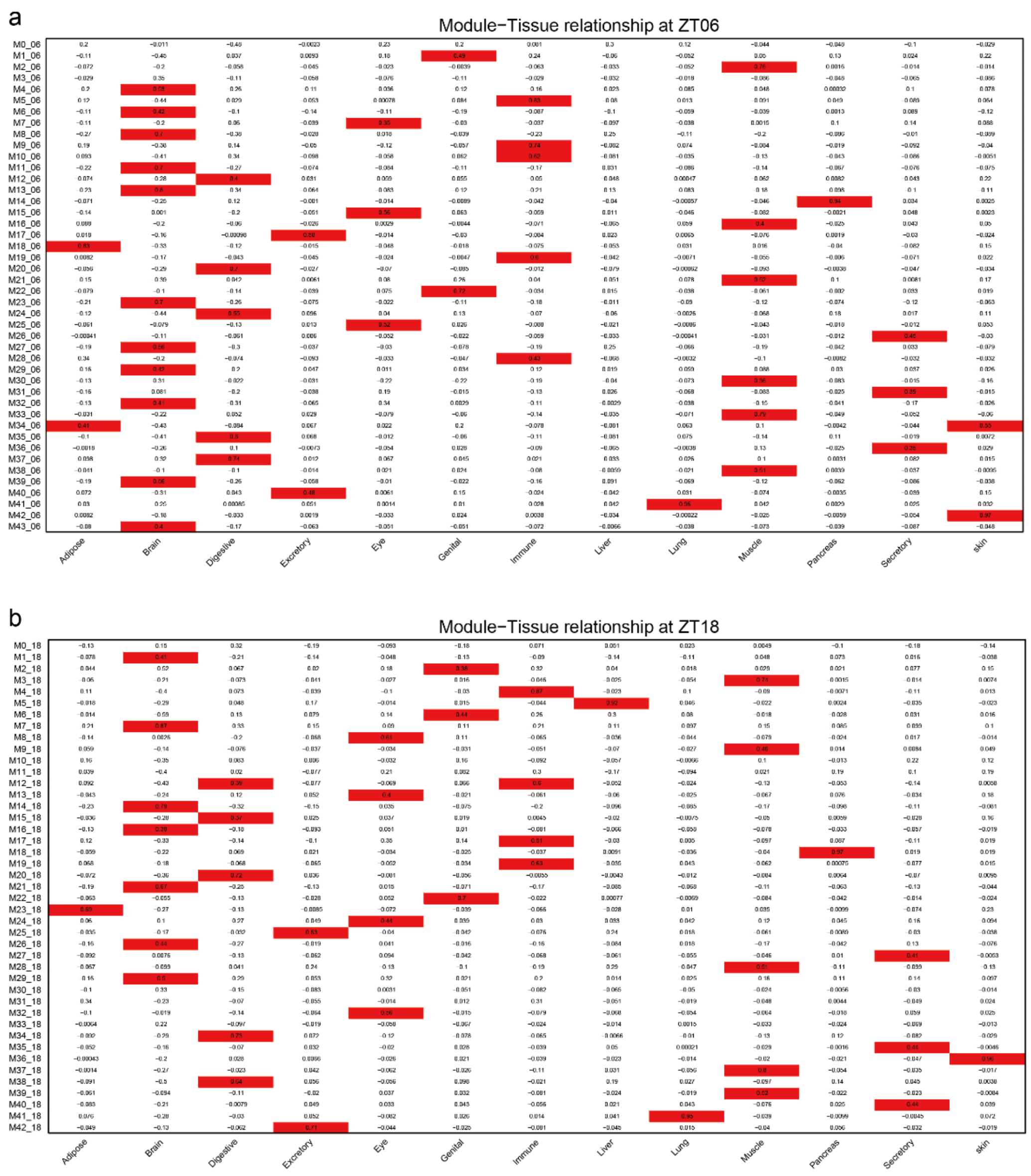

Supplementary Fig. 12 Relation between network modules and tissues. Pearson Correlation Coefficients were calculated by module eigengenes and tissue vectors (yes or no) to represent relation between modules and tissues at noon (**a**) and night (**b**).

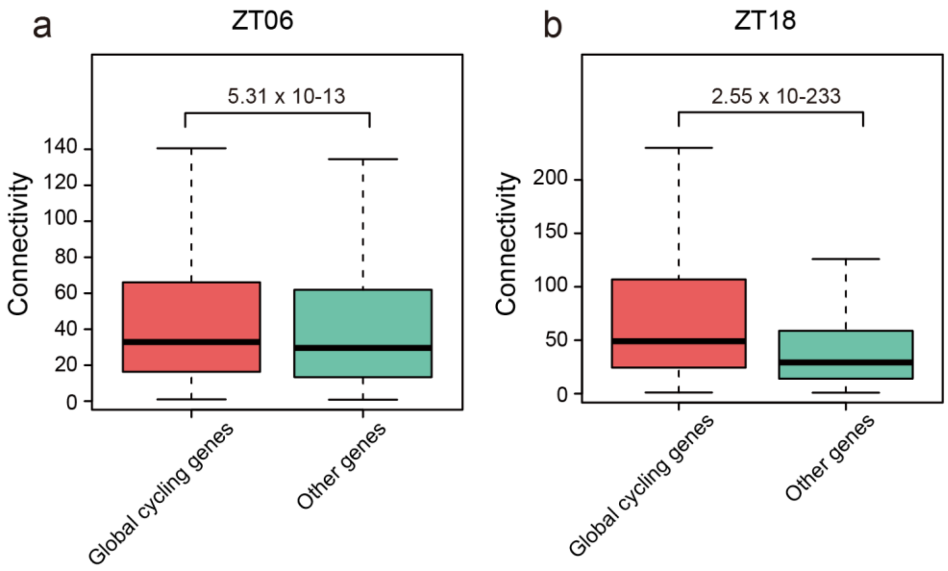

Supplementary Fig. 13 Global cycling genes are highly connected in both daytime (a) and nighttime (b) networks.

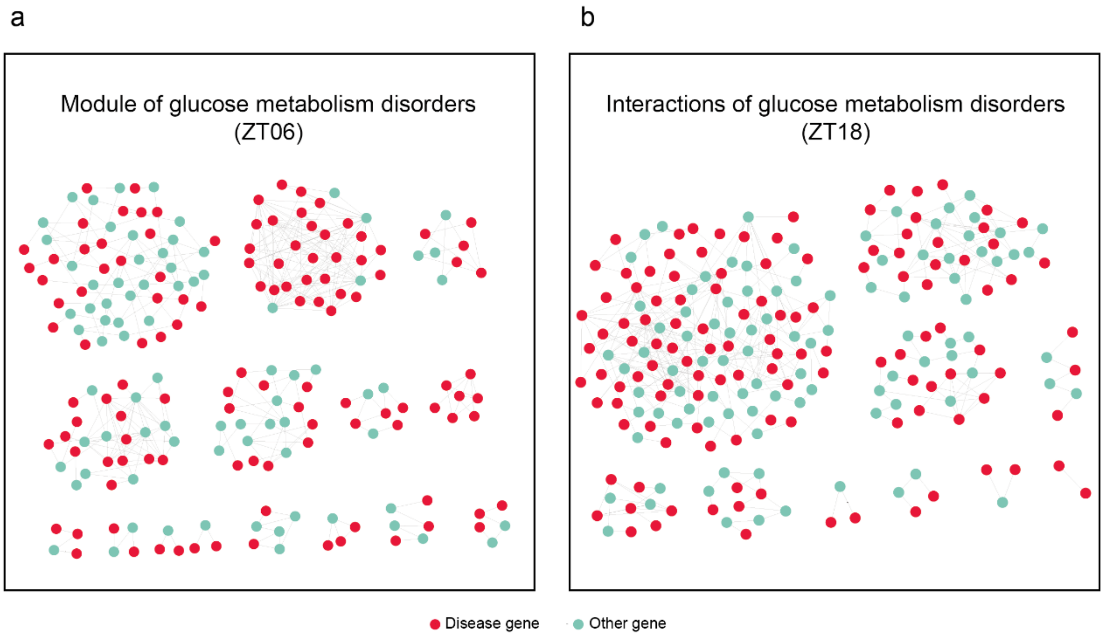

Supplementary Fig. 14 Distribution of glucose metabolism disorders genes in network at ZT06 (a) and ZT18 (b). Red nodes are disease genes and light blue nodes are genes that connect disease genes.

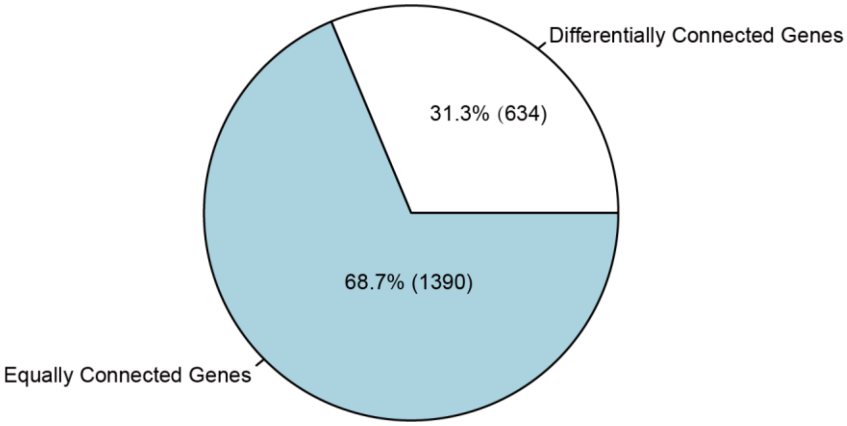

Supplementary Fig. 15 The proportion of differentially connected genes and equally connected genes in 2024 FDA drug target genes. >30% (634) of drug target genes are differentially connected between daytime and nighttime networks.

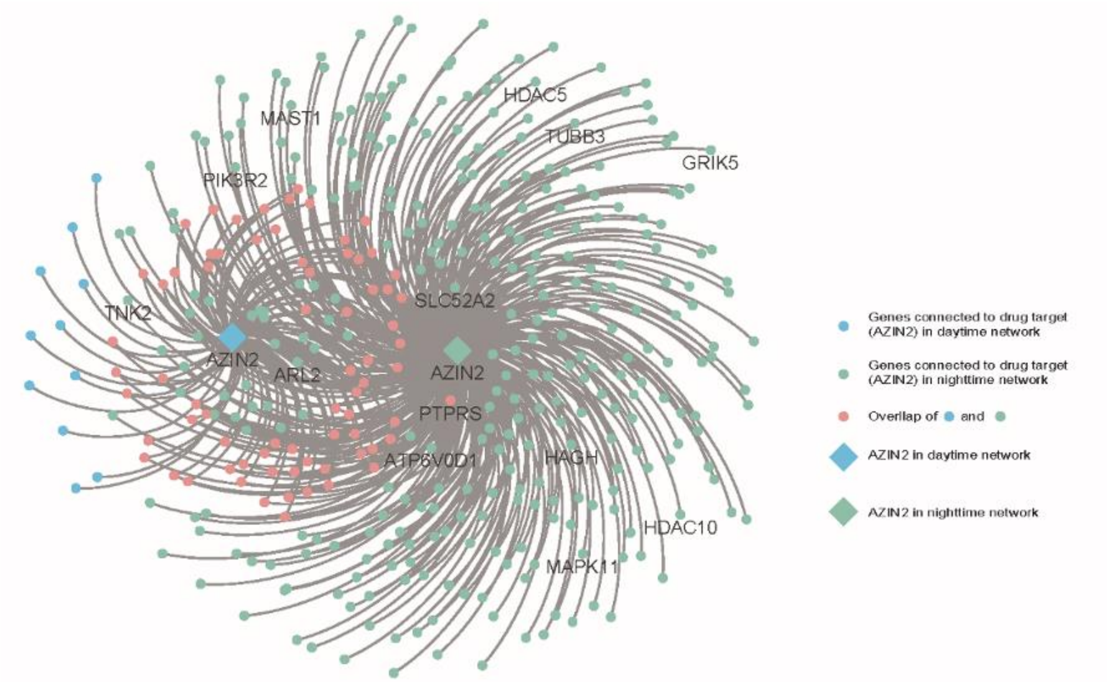

Supplementary Fig. 16 Network visualization of hub gene *AZIN2*. Differential connectivity of drug target gene *AZIN2* in daytime and nighttime networks.

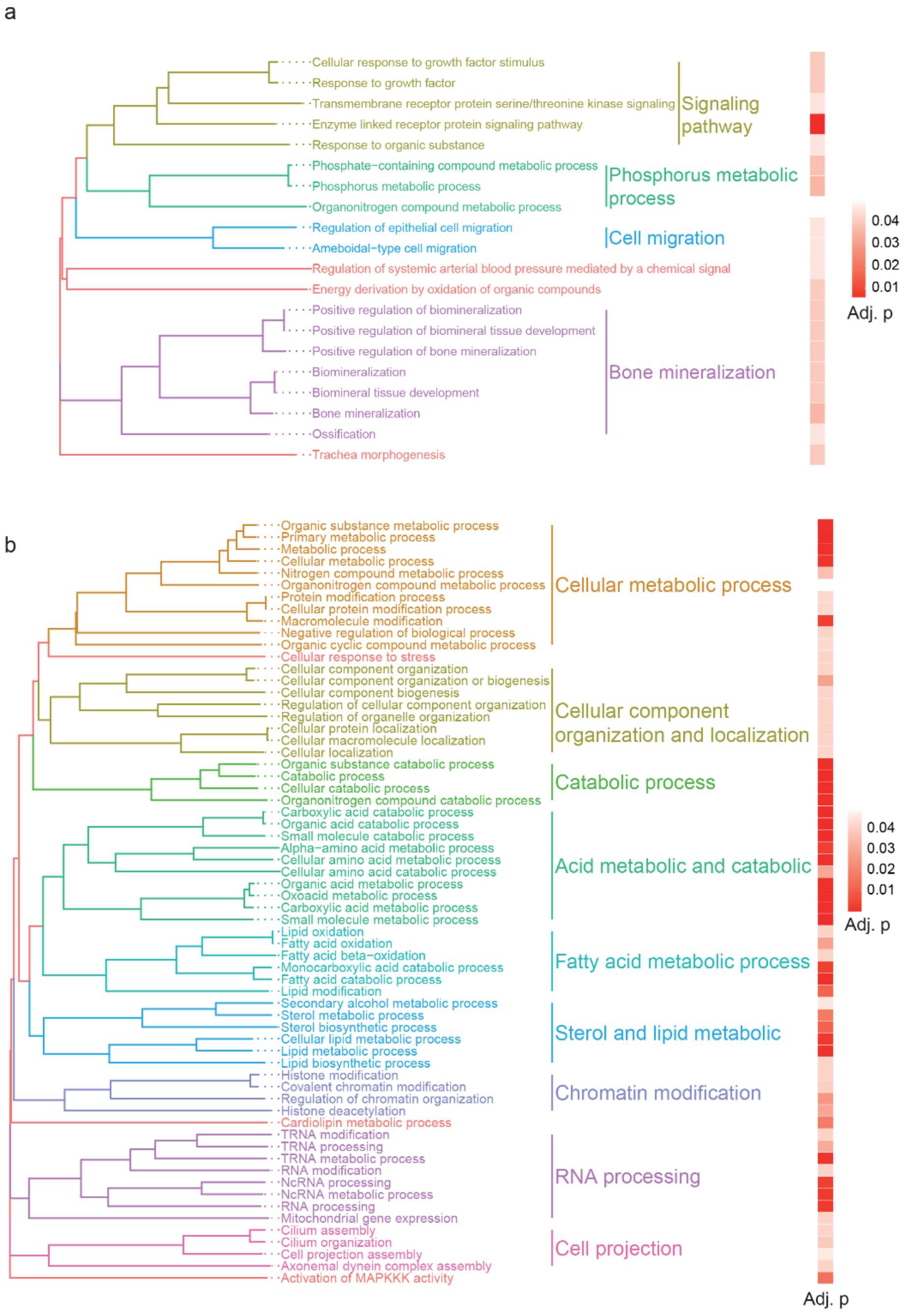

Supplementary Fig. 17 Comprehensive function annotations of differentially connected genes. a Biological pathways enriched for genes with less connectivity at ZT18. **b** Biological pathways enriched for genes with less connectivity at ZT06.

Supplementary code

```
library(WGCNA)

library(stringr)

options(stringsAsFactors = FALSE)

enableWGCNAThreads()


#### Generate body-wise network and modules using all samples from different times and tissues

## Remove IRI samples

identical(pdat$title, colnames(edat2))

rmSamples <- grep(pattern = "IRI", colnames(edat2) )

edat2 <- edat2[,-rmSamples]

pdat2 <- pdat[-rmSamples,]

identical(pdat2$title, colnames(edat2))


##log transformation

dat <- log10(edat2 + 1)


## Clustering

A=adjacency((dat),type="distance")

k=as.numeric(apply(A,2,sum))-1

Z.k=scale(k)

thresholdZ.k=-2.5

outlierColor=ifelse(Z.k<thresholdZ.k,"red","black")

traitColors=data.frame(tissues=pdat2$color, outlierColor)

sampleTree = hclust(as.dist(1-A), method = "average")

pdf("./sample_cluster.pdf")

plotDendroAndColors(sampleTree,

groupLabels=c("Tissues","Outlier"),
```

```
addGuide=TRUE,rowText = NULL,cex.rowText=0.6,
colors=traitColors,main="Sample dendrogram and trait heatmap")
dev.off()

## Set softpower
type = "signed"
# Choose a set of soft-thresholding powers
powers = c(c(1:10), seq(from = 12, to=30, by=2))
# Call the network topology analysis function
sft = pickSoftThreshold(t(dat),
powerVector = powers,
networkType = type,
corFnc="cor",
verbose = 5)

pdf("./estimate softpower.pdf",width=11.69,height = 8.27)
par(mfrow = c(1,2))
cex1 = 0.9;
# Scale-free topology fit index as a function of the soft-thresholding power
plot(sft$fitIndices[,1], -sign(sft$fitIndices[,3])*sft$fitIndices[,2],
xlab="Soft Threshold (power)",ylab="Scale Free Topology Model Fit,signed R^2",type="n",
main = "Scale independence");
text(sft$fitIndices[,1], -sign(sft$fitIndices[,3])*sft$fitIndices[,2],
labels=powers,cex=0.9,col="red");
# This line corresponds to using an R^2 cut-off of h
abline(h=0.8,col="red")

# Mean connectivity as a function of the soft-thresholding power
plot(sft$fitIndices[,1], sft$fitIndices[,5],
```

```
xlab="Soft Threshold (power)",ylab="Mean Connectivity", type="n",
main = "Mean connectivity")
text(sft$fitIndices[,1], sft$fitIndices[,5], labels=powers, cex=0.9,col="red")
dev.off()

## Build WGCNA network
softPower=16
print(softPower)
mergingThresh=0.25
pearson.net = blockwiseModules(t(dat),
corType="pearson",
maxBlockSize=16000,
networkType="signed",
power=softPower,
minModuleSize=50,
mergeCutHeight=mergingThresh,
numericLabels=TRUE,
saveTOMs=TRUE,
pamRespectsDendro=FALSE,
saveTOMFileBase="singed_pearsoncor_wgcna_15219genes756samplesFPKM")
save(pearson.net, file="singed_pearson_wgcna_15219genes756samplesFPKM.RData")

moduleLabelsAutomatic=pearson.net$colors
# Convert labels to colors for plotting
moduleColorsAutomatic = labels2colors(moduleLabelsAutomatic)
table(moduleColorsAutomatic)

# A data frame with module eigengenes can be obtained as follows
MEsAutomatic=pearson.net$MEs; dim(MEsAutomatic)
```

```
blocknumber=1

datColors=data.frame(moduleColorsAutomatic)[pearson.net$blockGenes[[blocknumber]],]

pdf(file="singed_pearson_wgcna_15219genes756samplesFPKM_dendrograms.pdf",width=11.69,
height = 8.27)

# Plot the dendrogram and the module colors underneath

plotDendroAndColors(pearson.net$dendrograms[[blocknumber]],

colors=datColors,

dendroLabels=FALSE,

hang =0.03,

addGuide=TRUE,

guideHang=0.05,

groupLabels=c("Module Colors"),

addTextGuide=TRUE,

main="Cluster Dendeogram")

dev.off()


MEs = pearson.net$MEs

MEs_col = MEs

colnames(MEs_col) = paste0("ME",
labels2colors(as.numeric(str_replace_all(colnames(MEs),"ME",""))))

MEs_col = orderMEs(MEs_col)

head(MEs_col)



pdf("singed_pearson_wgcna_15219genes756samplesFPKM_Eigengene_adjacency_heatmap.pd
f")

plotEigengeneNetworks(MEs_col, "Eigengene adjacency heatmap",

marDendro = c(3,3,2,4),marHeatmap = c(3,4,2,2),

plotDendrograms = T,

xLabelsAngle = 90)
```

```
dev.off()


### Get module genes

ZTtimes=unique(pdat2$time)

ZT.edat=lapply(ZTtimes,function(x){

return(edat2[,pdat2$title[pdat2$time==x]])

})

names(ZT.edat)=ZTtimes

lapply(ZT.edat,dim)


allmodules.genes=as.data.frame(pearson.net$colors)

allmodules.genes$module=labels2colors(allmodules.genes[,1])


geneAnno <- read.csv("I:/ensembl90-baboon-Panu2.0/ensmebl90-Panu2.0-geneData.txt",
sep="\t", header=TRUE, stringsAsFactors = FALSE)

head(geneAnno)

allmodules.genes$geneName <- geneAnno$Gene.name[match(row.names(allmodules.genes),
geneAnno$Gene.stable.ID)]

head(allmodules.genes)

core.clocks=c("CLOCK","NPAS2","ARNTL", "PER1", "PER2", "PER3",

"CRY1", "CRY2", "RORA","RORB", "RORC","NR1D1","NR1D2","DBP")

(allmodules.genes[allmodules.genes$geneName %in% core.clocks,])

write.table(allmodules.genes,

file="./signed_pearson_wgcna_module_genes.xls",

sep="\t", quote=FALSE)


getModuleGenes=function(allmodules.genes){

module.genes.list=list()

unique.module.v=unique(allmodules.genes$module)

unique.module.v.len=length(unique.module.v)
```

```
for(i in 1:unique.module.v.len){
temp.module=unique.module.v[i]
temp.module.genes=row.names(allmodules.genes)[allmodules.genes$module==temp.module]
module.genes.list[[i]]=temp.module.genes
names(module.genes.list)[i]=temp.module
}
return(module.genes.list)
}
module.genes.list=getModuleGenes(allmodules.genes)

nModule <- length(module.genes.list)
nSamples    <- ncol(edat2)

###Detect cycling module
##Detect cycling module by module mean expression
net.module.mean.dat=matrix(nrow=nModule,ncol=nSamples);
row.names(net.module.mean.dat)=names(module.genes.list);
colnames(net.module.mean.dat)=colnames(edat2)
for(i in 1:nModule){
temp.module=names(module.genes.list)[i]
net.module.mean.dat[temp.module,]=apply(edat2[module.genes.list[[temp.module]],],2,mean)
}


timepoints=unlist(strsplit(colnames(net.module.mean.dat),split="_ZT"))
timepoints=timepoints[seq(2,length(timepoints),2)]
timepoints=as.numeric(timepoints)
net.module.mean.dat=cbind(Module=row.names(net.module.mean.dat),net.module.mean.dat)
```

```
write.csv(net.module.mean.dat,

file="singed_pearson_wgcna_modulesMeans.csv",

row.names=FALSE)


meta2d(infile= "singed_pearson_wgcna_modulesMeans.csv",

outdir = "./",

filestyle = "csv",

cycMethod=c("ARS", "JTK", "LS"),

timepoints = timepoints,

outSymbol="Per24_",

minper=24,

maxper = 24,

nCores =4)


net.module.mean.meta2d.data=read.csv("./Per24_meta2d_singed_pearson_wgcna_modulesMea
ns.csv",

stringsAsFactors=FALSE)

colnames(net.module.mean.meta2d.data)

net.module.mean.meta2d.data[(net.module.mean.meta2d.data$JTK_BH.Q<0.05 &
net.module.mean.meta2d.data$JTK_adjphase!="NaN"),1]

net.module.mean.meta2d.data[(net.module.mean.meta2d.data$LS_BH.Q<0.05 &
net.module.mean.meta2d.data$LS_adjphase!="NaN"),1]

net.module.mean.meta2d.data[(net.module.mean.meta2d.data$meta2d_BH.Q<0.05 &
net.module.mean.meta2d.data$meta2d_phase!="NaN"),1]


# Plot heatmap for cycling modules

cycModule.dat <-
net.module.mean.meta2d.data[(net.module.mean.meta2d.data$JTK_BH.Q<0.05 &
net.module.mean.meta2d.data$JTK_adjphase!="NaN"),]

cycModule.dat <- cycModule.dat[order(cycModule.dat$JTK_adjphase),]

cycModule.dat

cycModule <- cycModule.dat$CycID
```

```
cycModule.mean <- net.module.mean.dat[cycModule,-1]

cycModule.mean <- apply(cycModule.mean,2,as.numeric); row.names(cycModule.mean) <-
cycModule

identical(colnames(cycModule.mean), pdat2$title)

plotdat <- aggregate(t(cycModule.mean), by=list(pdat2$time), mean )

row.names(plotdat ) <- plotdat [,1]; myname <- plotdat[,1]; plotdat    <- plotdat [,-1];

plotdat <- apply(plotdat,2,scale)

plotdat <- t(plotdat)

colnames(plotdat) <- myname

plotdat

pheatmap(plotdat,

border_color="grey60",

cellwidth = 20, cellheight =16,

treeheight_row=0,

cluster_cols=FALSE,

cluster_rows = FALSE,

color=colorRampPalette(c("green","black","red"))(100),

legend=TRUE,

main = "Module Mean expression heatmap",

filename="./Module Mean expression acorss 12 ZTtimes heatmap.pdf"

)



## Detect cycling module by module eigengene

net.MEs=pearson.net$MEs; net.MEs=t(net.MEs)

timepoints=unlist(strsplit(colnames(net.MEs),split="_ZT"))

timepoints=timepoints[seq(2,length(timepoints),2)]

timepoints=as.numeric(timepoints)

timepoints

#net.MEs=cbind(row.names(net.MEs),net.MEs)
```

```
row.names(net.MEs) = paste0("ME",
labels2colors(as.numeric(str_replace_all(row.names(net.MEs),"ME",""))))


write.csv(net.MEs,

file="singed_pearson_wgcna_MEs.csv")

meta2d(infile= "singed_pearson_wgcna_MEs.csv",

outdir = "./",

filestyle = "csv",

cycMethod=c("ARS", "JTK", "LS"),

timepoints = timepoints,

minper=24,

maxper = 24,

outSymbol="Per24_",

nCores =4)

net.module.MEs.meta2d.data=read.csv("./Per24_meta2d_singed_pearson_wgcna_MEs.csv",stri
ngsAsFactors=FALSE)


colnames(net.module.MEs.meta2d.data)

net.module.MEs.meta2d.data[(net.module.MEs.meta2d.data$JTK_BH.Q<0.05 &
net.module.MEs.meta2d.data$JTK_adjphase!="NaN"),1]

net.module.MEs.meta2d.data[(net.module.MEs.meta2d.data$LS_BH.Q<0.05 &
net.module.MEs.meta2d.data$LS_adjphase!="NaN"),1]

net.module.MEs.meta2d.data[(net.module.MEs.meta2d.data$meta2d_BH.Q<0.05 &
net.module.MEs.meta2d.data$meta2d_phase!="NaN"),1]

cycModule.dat <- net.module.MEs.meta2d.data[(net.module.MEs.meta2d.data$JTK_BH.Q<0.05
& net.module.MEs.meta2d.data$JTK_adjphase!="NaN"),]

cycModule.dat <- cycModule.dat[order(cycModule.dat$JTK_adjphase),]

cycModule <- cycModule.dat$CycID

cycModule.mean <- net.MEs[cycModule,]

cycModule.mean <- apply(cycModule.mean,2,as.numeric); row.names(cycModule.mean) <-
cycModule

identical(colnames(cycModule.mean), pdat2$title)
```

```
plotdat <- aggregate(t(cycModule.mean), by=list(pdat2$time), mean )

row.names(plotdat ) <- plotdat [,1]; myname <- plotdat[,1]; plotdat    <- plotdat [,-1];

plotdat <- apply(plotdat,2,scale)

plotdat <- t(plotdat)

colnames(plotdat) <- myname

row.names(plotdat) <- gsub("ME","", row.names(plotdat))

plotdat


pheatmap(plotdat,

border_color="grey60",

cellwidth = 20, cellheight =16,

treeheight_row=0,

cluster_cols=FALSE,

cluster_rows = FALSE,

color=colorRampPalette(c("green","black","red"))(100),

legend=TRUE,

main = "Module eigengene heatmap",

filename="./Module eigengene acorss 12 ZTtimes heatmap.pdf"

)


#### Generate the adjacency matrices of networks at different times

### The strength of the connection between gene pairs was obtained by the correlation
coefficient ### of gene expression in different tissues

rmSamples <- grep(pattern = "IRI", colnames(edat2) )

edat3 <- edat2[,-rmSamples]


output_adj <- function(x){

   dataExpr <- edat3[,str_detect(colnames(edat3),x)]

   ##Gene expression levels in different tissues were extracted at a given point in time

   dataExpr <- as.data.frame(t(dataExpr))
```

```
    adj <- adjacency(dataExpr,type = "signed",power = 16)
    save(adj,file = paste("adj_zt",x,".RData",sep = ""))
}
zt <- c("00","02","04","06","08","10","12","14","16","18","20","22")
sapply(zt,output_adj)

### Network indices including graph density, connectivity, clustering coefficient, centralization,
### heterogeneity and MAR are calculated by adjacency matrix
for(i in zt){
load(paste("./adj_zt",i,".RData",sep = ""))
net_fea <- fundamentalNetworkConcepts(adj_zt)
save(net_fea,file = paste("./net_fea_zt",i,".RData",sep = "") )
}

extract_netfea <- function(x,fea){
# This function load Network index data at a given time point.
load(paste("./net_fea_zt",x,".RData",sep = ""))
re <- net_fea[[fea]]
return(re)
}

Density <- sapply(zt,extract_netfea,"Density")
Centralization <- sapply(zt,extract_netfea,fea="Centralization")
Heterogeneity <- sapply(zt,extract_netfea,fea="Heterogeneity")
MAR <- sapply(zt,extract_netfea,fea="MAR")
ClusterCoef <- sapply(zt,extract_netfea,fea="ClusterCoef")
Connectivity <- sapply(zt,extract_netfea,fea="Connectivity")
median_clusterCoef <- apply(ClusterCoef,2,median)
median_Connectivity <- apply(Connectivity,2,median)
```

```
median_MAR <- apply(MAR,2,median)

ZT <- paste0("ZT",zt)

network_index <-
data.frame(Density,Centralization,Heterogeneity,median_Connectivity,median_clusterCoef,media
n_MAR,classification=c(rep("Day",6),rep("Night",6)),ZT=ZT)


### Compare networks at ZT06 and ZT18


## Gain gene expression matrix

data_zt06 <- edat3[,str_detect(colnames(edat3),"06")]

# The genes are in the column, the samples are in the row

data_zt06 <- as.data.frame(t(data_zt06))

data_zt18 <- edat3[,str_detect(colnames(edat3),"18")]

data_zt18 <- as.data.frame(t(data_zt18))


## Run the blockwise module detection for network zt ZT06

net_zt06 = blockwiseModules(data_zt06,

maxBlockSize=16000,

networkType="signed",

power=16,

minModuleSize=50,

mergeCutHeight=0.15,

numericLabels=TRUE,

saveTOMs=FALSE,

pamRespectsDendro=FALSE)

## Run the blockwise module detection for network zt ZT18

net_zt18 = blockwiseModules(data_zt18,

maxBlockSize=16000,

networkType="signed",

power=16,
```

```
minModuleSize=50,

mergeCutHeight=0.15,

numericLabels=TRUE,

saveTOMs=FALSE,

pamRespectsDendro=FALSE)


## Generate cluster dendrogram graphs

moduleColors1 <- labels2colors(net_06$colors)

moduleColors2 <- labels2colors(net_18$colors)

pdf("geneclusterplot06.pdf",width = 6, height = 4)


plotDendroAndColors(net_06$dendrograms[[1]],
moduleColors1[net_06$blockGenes[[1]]],main="Cluster Dendrogram of ZT06",

"Module colors",

dendroLabels = FALSE, hang = 0.2,

addGuide = TRUE, guideHang = 0.05,abHeight = seq(0.2,1,0.2))

pdf("geneclusterplot18.pdf",width = 6, height = 4)

plotDendroAndColors(net_18$dendrograms[[1]],
moduleColors2[net_18$blockGenes[[1]]],main="Cluster Dendrogram of ZT18",

"Module colors",

dendroLabels = FALSE, hang = 0.2,

addGuide = TRUE, guideHang = 0.05,abHeight = seq(0.2,1,0.2))


dev.off()


### The correlation between modules

a1 <-paste0("ME",0:43) ## Moduleas names for network at ZT06

a2 <- paste0("ME",0:42) ## Moduleas names for network at ZT18

moduleCor <- as.data.frame(cor(net_06$MEs,net_18$MEs))

moduleCor2 <- moduleCor[a1,a2]
```

```
textMatrix = signif(moduleCor2, 2)
rownames(textMatrix) <- paste(a1,"06",sep = "_")
colnames(textMatrix) <- paste(a2,"18",sep = "_")
dev.new()
par(mar = c(4,4.5,2,2))
labeledHeatmap(Matrix = moduleCor2,
xLabels = colnames(textMatrix),
yLabels = rownames(textMatrix),
colorLabels = FALSE,
colors = blueWhiteRed(50),
textMatrix = textMatrix,
setStdMargins = FALSE,
cex.text = 0.7,
zlim = c(-1,1),
main = paste("Module-Module eigengene similarity for zt06 and zt18"))
dev.off()

## Genes belong to different modules
ME_genes_zt06 <- tapply(net_06$colors,net_06$colors,names)
ME_genes_zt18 <- tapply(net_18$colors,net_18$colors,names)




jaccard_similarity <- function(x,y){
# This function calculates jaccard similarity for modules
return(length(intersect(x,y))/length(union(x,y)))
}
```

```
ME_jaccard <- matrix(nrow = 44,ncol = 43)
for(i in 1:length(ME_genes_zt06)){
for(j in 1: length(ME_genes_zt18)){
ME_jaccard[i,j] <- jaccard_similarity(ME_genes_zt06[[i]],ME_genes_zt18[[j]])
}
}

textMatrix2 = signif(ME_jaccard, 2)
dim(textMatrix2) = dim(ME_jaccard)
rownames(textMatrix2) <- paste("ME",names(ME_genes_zt06),"_06",sep = "")
colnames(textMatrix2) <- paste("ME",names(ME_genes_zt18),"_18",sep = "")
dev.new()
par(mar = c(4,4.5,2,2))
labeledHeatmap(Matrix = ME_jaccard,
xLabels = colnames(textMatrix2),
yLabels = rownames(textMatrix2),
colorLabels = FALSE,
colors = blueWhiteRed(50),
textMatrix = textMatrix2,
setStdMargins = FALSE,
cex.text = 0.7,
zlim = c(0,0.5),
main = paste("Module-Module jaccard similarity for zt06 and zt18"))
dev.off()

### Relation between module and tissues
## Gain sample information
tissue.dat <- read.csv("tissue.dat.csv")
tissue.dat <- tissue.dat[-21,]
```

```
br <- tissue.dat$broad_tissue
br_name <- names(table(br)) ## Broad tissues names
traitdata <- sapply(br_name,function(x){as.numeric(br == x)})
## The different tissues are represented by a vector consisting of 0,1
MEs_18 <- net_18$MEs
ME_18 <- MEs_18[,a2]
modTraitCor2 <- cor(ME_18, traitdata, use = "p")
modTraitP2 <- corPvalueStudent(modTraitCor2, 63)
modTraitP2 <- as.vector(modTraitP2)
modTraitP2 <- p.adjust(modTraitP2,method = "BH")
vetor_cor2 <- as.vector(modTraitCor2)
is_significant2 <- as.numeric(modTraitP2 < 0.05 & vetor_cor2 > 0)
dim(is_significant2) <- dim(modTraitCor2)
textMatrix3 = signif(modTraitCor, 2)
rownames(modTraitCor2) <- paste(a2,"18",sep = "_")
colnames(modTraitCor2) <- colnames(traitdata)
dev.new()
par(mar = c(4,4.5,2,2))
labeledHeatmap(Matrix = is_significant2,
xLabels = colnames(modTraitCor2),
yLabels = rownames(modTraitCor2),
colorLabels = FALSE,
colors = greenWhiteRed(50),
textMatrix = textMatrix3,
setStdMargins = FALSE,
cex.text = 0.7,
zlim = c(-1,1),
main = paste("Module-Tissue relationship at zt18"))
```

```
MEs_06 <- net_06$MEs

ME_06 <- MEs[,a1]

modTraitCor1 <- cor(ME_06, traitdata, use = "p")

modTraitP1 <- corPvalueStudent(modTraitCor1, 63)

modTraitP1 <- as.vector(modTraitP1)

modTraitP1 <- p.adjust(modTraitP1,method = "BH")

vetor_cor1 <- as.vector(modTraitCor1)

is_significant1 <- as.numeric(modTraitP1 < 0.05 & vetor_cor1 > 0)

dim(b) <- dim(modTraitP)

textMatrix4 = signif(modTraitCor1, 2)

rownames(modTraitCor1) <- paste(a1,"06",sep = "_")

colnames(modTraitCor1) <- colnames(traitdata)

dev.new()

par(mar = c(4,4.5,2,2))

labeledHeatmap(Matrix = is_significant1,

xLabels = colnames(modTraitCor1),

yLabels = rownames(modTraitCor1),

colorLabels = FALSE,

colors = greenWhiteRed(50),

textMatrix = textMatrix4,

setStdMargins = FALSE,

cex.text = 0.7,

zlim = c(-1,1),

main = paste("Module-Tissue relationship at zt06"))


dev.off()


### Define connectivity differential genes

d_zt06 <- Connectivity[,4]
```

```
d_zt18 <- Connectivity[,10]
logFC <- log2(d_zt06/d_zt18)
up_zt06 <- names(logFC[logFC > 1])
up_zt18 <- names(logFC[logFC < -1])
degree_differntial_genes <- union(up_zt06,up_zt18)

### Euclidean distance between gene pairs is calculated from the vector of genes
### in the adjacency matrix
eucil_dist <- function(x,y){
x <- as.vector(x)
y <- as.vector(y)
return(sqrt(sum((x-y)^2)))
}
pair_eucildist <- function(adj1,adj2,n){
re <- vector(mode = "numeric",length = n)
for(i in 1:n){
re[i] <- eucil_dist(adj1[i,],adj2[i,])
}
return(re)
}
eucil_dist <- pair_eucildist(adj_zt06,adj_zt18,15219)
names(eucil_dist) <- rownames(adj_zt06)
```
