# Supplementary material for: Gene networks under circadian control exhibit diurnal organization in primate organs
Source: Commun Biol. 2022 Jul 29;5:764. doi: 10.1038/s42003-022-03722-0 (PMC9334736; doi:10.1038/s42003-022-03722-0)
Supplement: Supplementary file 2 — Description of Additional Supplementary Files [file 42003_2022_3722_MOESM2_ESM.pdf]

Description of Additional Supplementary Files

File name: Supplementary Data 1
Description: Detailed information of global cycling genes in baboon and mouse.

File name: Supplementary Data 2
Description: Rhythmicity of core circadian gene-gene interactions.

File name: Supplementary Data 3
Description: Detailed information of body-wide network modules and their rhythmicity.

File name: Supplementary Data 4
Description: Network indices at different time points.

File name: Supplementary Data 5
Description: Details of network modules at ZT06 and ZT18.

File name: Supplementary Data 6
Description: Consensus modules and their relationship with different tissues.

File name: Supplementary Data 7
Description: Biological pathways enrichment for genes which have less connectivity at ZT06 and ZT18.

File name: Supplementary Data 8
Description: Conserved COVID-19 related cycling genes among human, mouse and baboon.

File name: Supplementary Data 9
Description: Gene sets involved in circadian rhythms, viral process and immune response.
