# Supplementary material for: Gene networks under circadian control exhibit diurnal organization in primate organs
Source: Commun Biol. 2022 Jul 29;5:764. doi: 10.1038/s42003-022-03722-0 (PMC9334736; doi:10.1038/s42003-022-03722-0)
Supplement: Supplementary file 12 — Reporting summary [file 42003_2022_3722_MOESM12_ESM.pdf]

nature research

Corresponding author(s): Guang-Zhong Wang

Last updated by author(s): June 24, 2022

Reporting Summary

Nature Research wishes to improve the reproducibility of the work that we publish. This form provides structure for consistency and transparency in reporting. For further information on Nature Research policies, see our Editorial Policies and the Editorial Policy Checklist.

Please do not complete any field with "not applicable" or n/a. Refer to the help text for what text to use if an item is not relevant to your study.
For final submission: please carefully check your responses for accuracy; you will not be able to make changes later.

Statistics

For all statistical analyses, confirm that the following items are present in the figure legend, table legend, main text, or Methods section.

n/a	Confirmed	
☐	☒	The exact sample size (*n*) for each experimental group/condition, given as a discrete number and unit of measurement
☐	☒	A statement on whether measurements were taken from distinct samples or whether the same sample was measured repeatedly
☐	☒	The statistical test(s) used AND whether they are one- or two-sided<br>*Only common tests should be described solely by name; describe more complex techniques in the Methods section.*
☐	☒	A description of all covariates tested
☐	☒	A description of any assumptions or corrections, such as tests of normality and adjustment for multiple comparisons
☐	☒	A full description of the statistical parameters including central tendency (e.g. means) or other basic estimates (e.g. regression coefficient) AND variation (e.g. standard deviation) or associated estimates of uncertainty (e.g. confidence intervals)
☐	☒	For null hypothesis testing, the test statistic (e.g. *F*, *t*, *r*) with confidence intervals, effect sizes, degrees of freedom and *P* value noted<br>*Give P values as exact values whenever suitable.*
☒	☐	For Bayesian analysis, information on the choice of priors and Markov chain Monte Carlo settings
☐	☒	For hierarchical and complex designs, identification of the appropriate level for tests and full reporting of outcomes
☐	☒	Estimates of effect sizes (e.g. Cohen's *d*, Pearson's *r*), indicating how they were calculated

Our web collection on statistics for biologists contains articles on many of the points above.

Software and code

Policy information about availability of computer code

Data collection	Expression data were downloaded from NCBI's Gene expression Omnibus (https://www.ncbi.nlm.nih.gov/geo/), with GEO accession number: GSE98965.
Data analysis	We applied JTK_CYCLE (version 3) to identify the oscillations of genes, gene-gene interactions and modules, then utilized WCGNA (version1.69) to build weighted co-expresion network in R (version 4.0.2)

For manuscripts utilizing custom algorithms or software that are central to the research but not yet described in published literature, software must be made available to editors and reviewers. We strongly encourage code deposition in a community repository (e.g. GitHub). See the Nature Research guidelines for submitting code & software for further information.

Data

Policy information about availability of data

All manuscripts must include a data availability statement. This statement should provide the following information, where applicable:

- Accession codes, unique identifiers, or web links for publicly available datasets
- A list of figures that have associated raw data
- A description of any restrictions on data availability

Gene expression was downloaded from NCBI's Gene expression Omnibus, with GEO accession number: GSE98965. Protein-protein interaction data were downloaded from STRING database (https://string-db.org/) and HIPPIE database (http://cbdm-01.zdv.uni-mainz.de/~mschaefer/hippie/). Genetic interactions were obtained from BioGRID database (https://thebiogrid.org/) and functional relevant interactions were downloaded from KEGG pathway database (https://www.genome.jp/kegg/pathway.html). The 332 COVID-19 interacting proteins were from previous study (ref 36).

Field-specific reporting

Please select the one below that is the best fit for your research. If you are not sure, read the appropriate sections before making your selection.

☒ Life sciences ☐ Behavioural & social sciences ☐ Ecological, evolutionary & environmental sciences

For a reference copy of the document with all sections, see nature.com/documents/nr-reporting-summary-flat.pdf

Life sciences study design

All studies must disclose on these points even when the disclosure is negative.

Sample size	Sample size was already predetermined since dataset has already been published, 756 samples were included in the analysis
Data exclusions	Samples from iris are excluded because of the lack of one time point
Replication	In the estimation of the proportion of rhythmic interactions, 100 replications were performed to obtain an unbiased assessment.
Randomization	In the case of rhythmic interaction identification, we select 10,000 gene pairs randomly from different group of genes, such as all expressed protein-coding genes, highly expressed genes, gene pairs from protein-protein interactions, genetic interactions and KEGG pathways.
Blinding	It is not applicable in our work

Reporting for specific materials, systems and methods

We require information from authors about some types of materials, experimental systems and methods used in many studies. Here, indicate whether each material, system or method listed is relevant to your study. If you are not sure if a list item applies to your research, read the appropriate section before selecting a response.

Materials & experimental systems

n/a	Involved in the study	
☒	☐	Antibodies
☒	☐	Eukaryotic cell lines
☒	☐	Palaeontology and archaeology
☒	☐	Animals and other organisms
☒	☐	Human research participants
☒	☐	Clinical data
☒	☐	Dual use research of concern

Methods

n/a	Involved in the study	
☒	☐	ChIP-seq
☒	☐	Flow cytometry
☒	☐	MRI-based neuroimaging

April 2020
